# Identification of differentially expressed genes in sunflower (*Helianthus annuus*) leaves and roots under drought stress by RNA sequencing

**DOI:** 10.1186/s40529-017-0197-3

**Published:** 2017-10-25

**Authors:** Chunbo Liang, Wenjun Wang, Jing Wang, Jun Ma, Cen Li, Fei Zhou, Shuquan Zhang, Ying Yu, Liguo Zhang, Weizhong Li, Xutang Huang

**Affiliations:** 1 Heilongjiang Academy of Agricultural Sciences Postdoctoral Programme, Xuefu Road 368, Harbin, 150086 People’s Republic of China; 2 Institute of Industrial Crops, Heilongjiang Academy of Agricultural Sciences, Harbin, 150086 People’s Republic of China; 3 Crop Research and Breeding Center of Land-Reclamation of Heilongjiang Province, Harbin, 150036 People’s Republic of China

**Keywords:** Digital gene expression, Drought stress, Leaf, Sunflower (*Helianthus annuus* L.), Root

## Abstract

**Background:**

Sunflower is recognized as one of the most important oil plants with strong tolerance to drought in the world. In order to study the response mechanisms of sunflower plants to drought stress, gene expression profiling using high throughput sequencing was performed for seedling leaves and roots (sunflower inbred line R5) after 24 h of drought stress (15% PEG 6000). The transcriptome assembled using sequences of 12 samples was used as a reference.

**Results:**

805 and 198 genes were identified that were differentially expressed in leaves and roots, respectively. Another 71 genes were differentially expressed in both organs, in which more genes were up-regulated than down-regulated. In agreement with results obtained for other crops or from previous sunflower studies, we also observed that nine genes may be associated with the response of sunflower to drought.

**Conclusions:**

The results of this study may provide new information regarding the sunflower drought response, as well as add to the number of known genes associated with drought tolerance.

**Electronic supplementary material:**

The online version of this article (doi:10.1186/s40529-017-0197-3) contains supplementary material, which is available to authorized users.

## Background

Drought is one of the most serious ecological problems challenging mankind. Statistically, of all natural insults, drought stress has the highest impact on crop yields worldwide and its impact may equal the total impact of all other abiotic stresses combined (Chen et al. 2009). Due to the magnitude of drought’s influence, improving crop drought tolerance is an urgent priority. Modern molecular biological technologies have greatly facilitated elucidation of plant response mechanisms to water stress, which in turn are relevant to understanding the genetic basis of drought tolerance of plants. Ultimately, identification of drought tolerance genes should inform breeding strategies for eventual creation of drought resistant sunflower hybrids.

Sunflower (*Helianthus annuus*) is the fourth largest source of vegetable oil in the world. In 2016, the global planting area of this crop was greater than 24,970,000 hm^2^ and total output approached 45,750,000 tons (the Food and Agriculture Organization of the United Nations, FAO). China is one of the most important producers and consumers of sunflower in the world. Since sunflower plants are somewhat tolerant to drought, poor soil conditions and salt, sunflower is suitable for cultivation in most areas of Heilongjiang Province. Consequently, improved sunflower drought tolerance would allow cultivation of this crop across an even larger area than currently utilized, extending to the mid-western arid and semi-arid middle- and low-yielding fields of this province. By first studying sunflower varieties most resistant to drought, with characterization of drought resistance genes and molecular mechanisms underlying drought tolerance, improved drought-tolerant sunflower might then be effectively developed.

In recent years, researchers have paid increasing attention to the molecular biology underlying drought tolerance mechanisms in order to improve sunflower drought tolerance. For example, Liu et al. (2003) analyzed the expression of five drought-induced genes in roots, stems and leaves of sunflower using differential display PCR and real-time quantitative PCR. Using a different approach, Diaz-Martin et al. (2005) cloned a drought-related DREB transcription factor, HaDREB2, in sunflower using gene interaction analyses. Meanwhile, Roche et al. (2007) analyzed the expression of genes associated with metabolism and signal transduction in leaves and immature embryos of sunflower using a cDNA array. Subsequently, 409 differentially expressed genes (DEGs) were identified, of which 82 were organ specific and induced by drought stress. In addition, Kane and Risesberg (2007) identified candidate sunflower drought and salt tolerance genes using selective screening and discovered 17 genes that were induced by each of these stresses. Subsequently, Cheng et al. (2009) transformed the drought and salt resistance gene P5CS into sunflower and obtained six transformed buds which were resistant to Kan, thus showing that successful gene transfer and expression had occurred; however, no fertile transgenic plants were obtained. Soon thereafter, Sauca et al. (2011) introduced the same drought resistance gene into cultivated sunflower inbred lines using an embryo rescue technique. More recently, Yi et al. (2013) demonstrated that a betaine aldehyde dehydrogenase (BADH) gene is induced by drought and salt stress in sunflower.

Moreover, due to the lack of a complete sunflower transcriptome, the study of molecular mechanisms underlying drought resistance has mainly focused on several independent genes instead of focusing on broader stress-induced genetic response networks. Because tolerance to drought is a complex phenotypic trait controlled by polygenes, it would not be useful to research any single gene or single class of genes to understand molecular mechanisms underlying drought tolerance. Fortunately, transcriptome profiling and digital gene expression (DGE) analysis using high throughput sequence technology have facilitated discovery of DEGs under drought stress without relying on genome sequence information. Although annotation of DEGs is challenging without a genome sequence, the information acquired from DGE profiling could nonetheless describe the stress response mechanism to a certain extent.

In this study, DGE profiling of leaves and roots of sunflower (inbred line R5) in response to drought stress was conducted using Illumina high throughput sequencing technology. The DEGs that were subsequently described here should lay the foundation for research of molecular mechanisms of sunflower drought tolerance. Moreover cloning and expression analysis of DEGs identified in this study may provide several candidate genes and information to improve sunflower drought stress tolerance using genetic engineering technology.

## Methods

### Plant growth and stress treatments

The sunflower line R5 was used in this study. R5 seeds were provided by the Industrial Crops Institute of Heilongjiang Academy of Agricultural Sciences. Sunflower seeds were rinsed with sterilized distilled water three times and allowed to germinate in Petri dishes for 72 h at 28 °C in darkness. The germinated seeds were transferred into pots containing nutrients, soil and vermiculite (1:1). Seedlings were grown in an artificial climate chamber with 16 h light and 8 h darkness (22 °C) with 70% relative humidity. Seedlings at the four-leaf stage showing appropriate growth status were rinsed with sterilized distilled water and placed into culture bottles filled with 15% PEG in water for 24 h to serve as drought stressed plants. Control plants (distilled water only) were grown in parallel and collected at the same time points. The process was repeated in triplicate, where each replicate was biologically and temporally independent.

### Sample preparation and RNA isolation

The leaves and roots of drought-stressed and control groups were harvested for gene expression analysis. The tissue samples were immediately frozen in liquid nitrogen and stored at − 80 °C. Total RNA was isolated separately from the leaves and roots of sunflower seedlings using TRIzol Reagent (Tiangen, Beijing, China) following the manufacturer’s protocols. Yield and quality of the RNA samples were determined using a NANODROP 2000 spectrophotometer (Thermo Fisher Scientific, USA). Since no genome sequence has yet been generated for sunflower, a portion of the total RNA from each of 12 samples studied here was pooled for construction of a library for use as a reference to validate transcriptome sequencing results presented here.

### Library preparation and sequencing

Total RNA preparations from 13 samples (including one pool of all samples and three samples from each of four sample types: leaves from PEG-treated plants, roots from PEG-treated plants, leaves from control plants, roots from control plants) were used for mRNA isolation and concentration using magnetic oligo (dT) beads. The mRNA was broken into short segments by adding fragmentation buffer. With mRNA as the template, first-strand cDNA was synthesized by adding random hexamers and second-strand cDNA was synthesized by adding buffer, dNTPs, DNA polymerase I and RNase H. Double-stranded cDNA was purified using AMPure XP beads. The ends of purified double-stranded cDNA were repaired, A-tails were added and overlapping sequences were joined. Next, AMPure XP beads were used for fragment size selection. Finally, the cDNA libraries were constructed using PCR amplification and products were purified using AMPure XP beads. Qubit 2.0 was used to perform a preliminary quantitation of the cDNA library. Each library was diluted to 1.5 ng/µL and an Agilent 2100 Bioanalyzer was used to analyze the insert size range of the cDNA library. Quantitative PCR (qPCR) was used to analyze the effective concentration of each library. In order to ensure the quality of each library, the effective concentration of library must be > 2 nM. According to the effective concentration and data quantification, the various libraries were pooled and sequenced using Illumina HiSeq/MiSeq.

### Sequencing data analysis

In order to ensure the quality of downstream analyses, the raw reads obtained from sequencing were filtered. Clean reads were obtained after removing from the raw sequence data the reads containing only adapters, reads with a content of N > 10% and low quality reads. All downstream analyses were based on clean reads with high quality.

Trinity software (v2012-10-05) was used for splicing of transcripts (Grabherr et al. 2011). The databases used for gene annotation in this study included: nr (NCBI non-redundant protein sequences), nt (NCBI nucleotide sequences), Pfam (Protein family), KOG/COG (euKaryotic Ortholog Groups/Clusters of Orthologous Groups of proteins), Swiss-Prot (a manually annotated and reviewed protein sequence database), KEGG (Kyoto Encyclopedia of Genes and Genomes) an GO (Gene Ontology). MISA (1.0) software was used to detect simple sequence repeats (SSRs) in the unigenes and Primer 3 (2.3.5) was used to design primers to characterize SSR markers. Clean DEG sequence reads were mapped to the transcripts by RSEM (Li and Dewey 2011) and were used to obtain the read count for each sample that mapped to each gene. Finally, the read count number was converted to FPKM (expected number of Fragments Per Kilobase of transcript sequence per Millions base pairs sequenced) (Trapnell et al. 2010). DESeq (Anders et al. 2010) was used to analyze differential gene expression. The screening threshold was padj < 0.05. This method was based on a negative binomial distribution model; the read count of gene i in the sample of j was designated K_ij_. The P values were adjusted using the Benjamini and Hochberg method. The corrected *P* value of 0.005 and log2 (fold change) of 1 were set as thresholds for significance for scoring of differential expression. The method of GO enrichment analysis in this study was GOseq (Young et al. 2010). KOBAS (2.0) software was used to test the statistical enrichment of differential expression of genes in the KEGG pathway (Mao et al. 2005).

### Quantitative real-time PCR (qRT-PCR) analysis

Five genes (comp9755_c0, comp11967_c0, comp29815_c0, comp36138_c0 and comp72325_c0) which have been identified may be related to drought stress response were validated by quantitative real-time PCR (qRT-PCR) using the same RNA samples as in the DGE library construction. Leaves and roots were removed from the freezer and ground in liquid nitrogen. Six pairs of gene-specific primers (five candidate genes and one actin gene) were designed based on target gene sequences using Primer Express software (Additional file 1: Table S1). First-strand cDNA synthesis and qRT-PCR were carried out using a PrimeScript™ RT reagent Kit with gDNA Eraser (TaKaRa), SYBR Premix Ex Taq™ II (Tli RNaseH Plus) and ROX plus (TaKaRa). Each PCR (18 µL) contained 10 µL 2 × Real-Time PCR Mix, 5 µM of each primer and diluted cDNA. The thermal cycling conditions were 95 °C for 30 s followed by 40 cycles of 5 s at 95 °C and 40 s at 60 °C. All reactions were performed in triplicate, including the non-template controls. The relative expression levels were calculated as 2 ^−(△Ct of treatment^ ^− △Ct of control)^.

## Result

### Results of sunflower transcriptome sequencing

To identify genes that are differentially expressed in response to drought stress, 12 samples [leaves of plants treated with PEG (three samples), roots of plants treated with PEG (three samples), leaves of control plants (three samples) and roots of control plants (three samples)] were mixed for the construction of a library for transcriptome sequencing to serve as a reference DGE sequence library. In this study, a total of 62,252 unigenes were obtained by merging overlapping transcript fragments for each locus. By analyzing the length of transcripts and unigenes, we found that the numbers of unigenes of lengths of 200–500 bp, 0.5–1 kb, 1–2 kb or greater than 2 kb were 37,645, 12,190, 8714 and 3703, respectively. The average length of all gene fragments was 390 bp. All unigenes were annotated using NR, NT, KO, Swiss-Proto, PFAM, GO, KOG databases. The results demonstrated that 39,356 unigenes could be annotated using at least one database, accounting for 62.33% of the total unigenes observed. Of these, 4152 unigenes could be annotated using all seven databases, which accounted for 6.66% of the total number of unigenes (Table 1), the detailed results of annotation is listed in Additional file 2: Table S2. After annotation using the GO database, successfully annotated genes could be categorized into 47 sub-groups from three main categories (biological process, cellular component and molecular function) (Additional file 3: Table S3). The results of this study should provide abundant gene resources for future studies focusing on the molecular biology of sunflower.

**Table 1:** Success rate statistics of gene annotation

Term	Number of unigenes	Percentage
Annotated in NR	36,286	58.28
Annotated in NT	12,683	20.37
Annotated in KO	12,043	19.34
Annotated in Swiss Prot	26,583	42.7
Annotated in PFAM	25,009	40.17
Annotated in GO	28,579	45.9
Annotated in KOG	14,038	22.55
Annotated in all databases	4152	6.66
Annotated in at least one database	39,356	63.22
Total	62,252	100

NR, NT, KO, Swiss Prot, PFAM, GO and DOG indicate the database of NCBI non-redundant protein sequences, NCBI, nucleotide sequences, Kyoto Encyclopedia Genes and Genomes Orthology, a manually annotated and reviewed protein sequence database, Protein family, Gene ontology and euKaryotic Orthology groups

In this study, 4,283,598 bp nucleotides belonging to 62,252 unigenes were analyzed for SSR calling. Subsequently, 8808 SSR sequences were found in 7441 unigenes of which 1120 unigenes contained more than one SSR sequence and 507 SSR sequences were present in compound formations. The primers of these SSR sequences are listed in Additional file 4: Table S4.

### Identification of DEGs in sunflower leaves and roots in response to drought stress

**Fig. 1:**
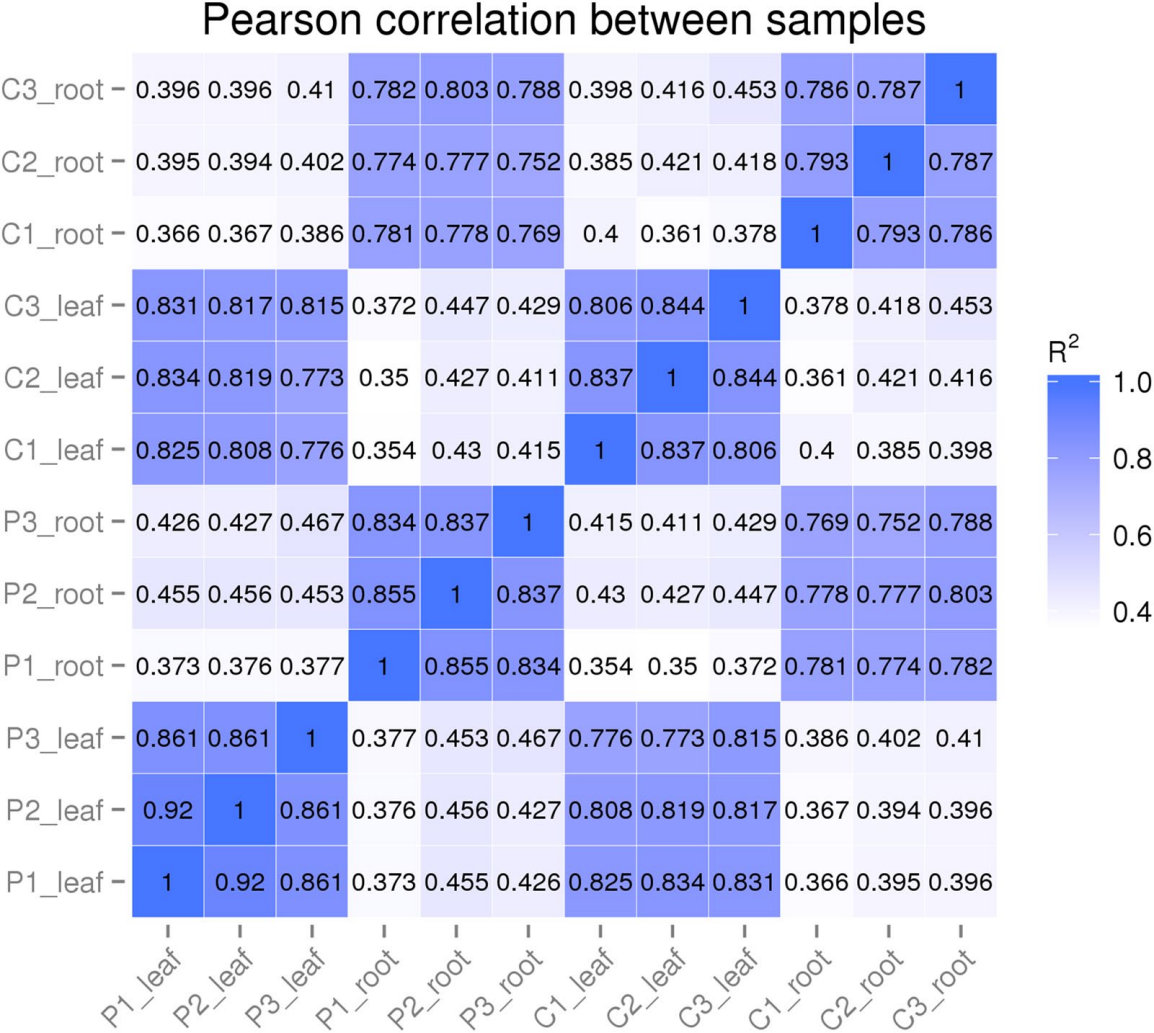
Pearson correlation between samples. C represents the control group; P represents the PEG-treated group. R^2^ represents the correlation coefficient. The darker the blue background, the greater the correlation coefficient

The results of correlation analysis of three replicates for each treatment showed that the correlation index between the replicates ranged from 0.786 to 0.920 (Fig. 1), which indicated that the sequencing data were reliable and could be used for further analysis.

**Fig. 2:**
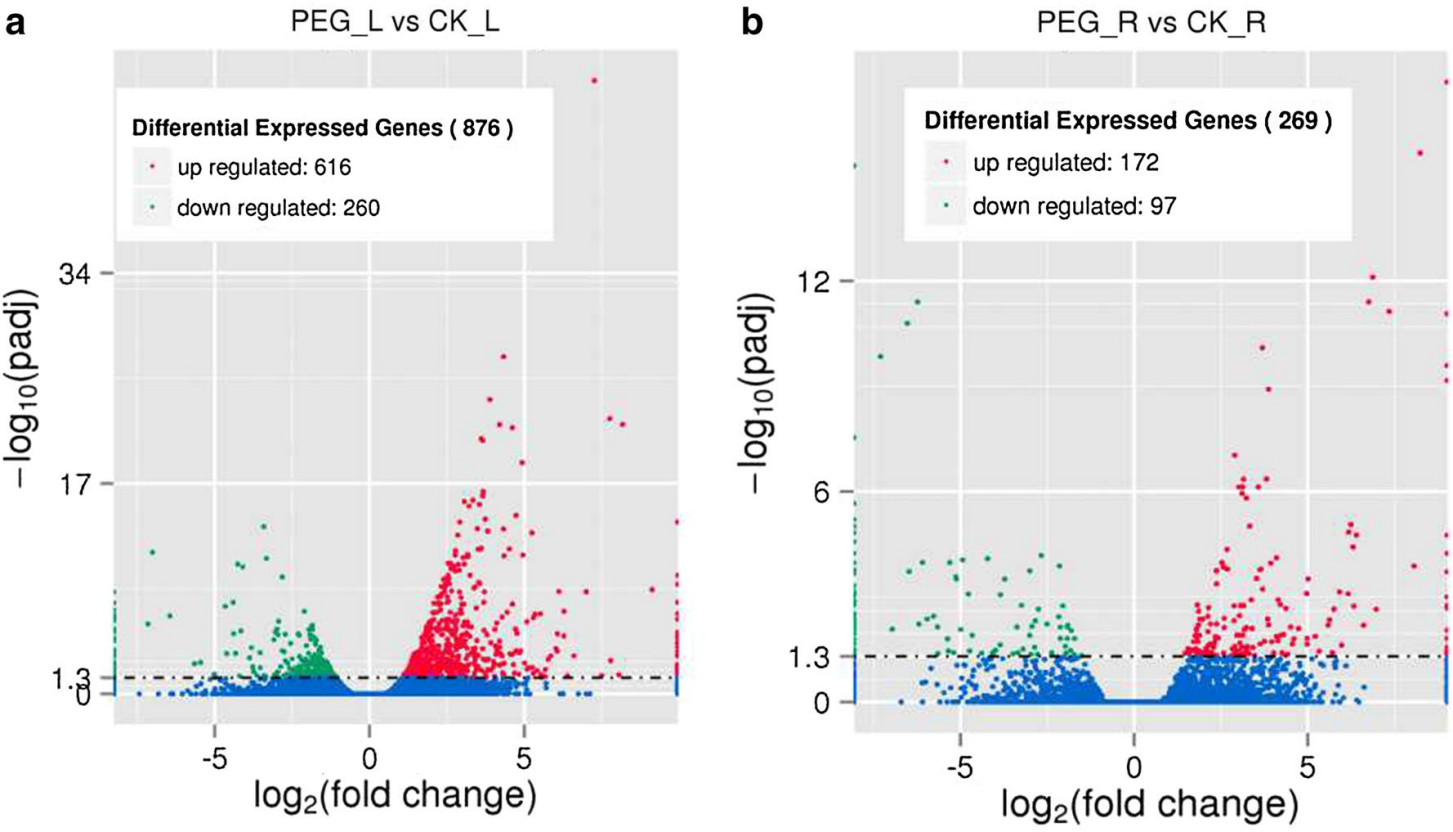
Volcano plot of significantly differently expressed genes in leaves and roots under drought stress. Red and green dot represent the up-regulated and down-regulated genes, respectively. **a** Differential expressed genes in the leaves under PEG treatment (PEG _L) and control group (CK_L). **b** Differential expressed genes in the roots under PEG treatment (PEG _R) and control group (CK_R)

In this study, 876 and 269 genes were differentially expressed between control and drought-stressed plants in their leaves and roots, respectively. Next, gene expression profiles were compared between leaves and roots under drought stress. Under drought stress, 805 and 198 DEGs were identified to be leaf-specific and root-specific, respectively, and 71 of these genes were differentially expressed both in leaves and roots. Among these DEGs, 616 and 172 genes were up-regulated during drought stress in leaves and roots, respectively, while 260 and 97 genes were down-regulated in leaves and roots, respectively (Fig. 2). Of the 616 up-regulated genes in leaves, 26 and 415 genes could be annotated in sunflower and other plant species, respectively, while 175 genes could not be annotated. Of the 260 down-regulated genes in leaves, 14 and 191 genes could be annotated in sunflower and other plant species, respectively. In the 172 up-regulated genes in roots, 5 and 110 genes could be annotated in sunflower and other plant species, respectively, while 57 genes could not be annotated. Of the 97 down-regulated genes in roots, three and 60 genes could be annotated in sunflower and other plant species, respectively, while 34 genes could not be annotated.

In response to drought, both leaves and roots showed a greater number of up-regulated genes than down-regulated genes. Some stress-induced genes were regulated in a tissue-specific manner, which indicates that these genes may play roles as part of distinct mechanisms for coping with drought stress. Another 71 genes exhibited the same pattern of expression in leaves versus roots, indicating that overlaps exist at the transcriptional level between leaves and roots in their response to drought stress.

### Functional categorization of stress-regulated genes

**Fig. 3:**
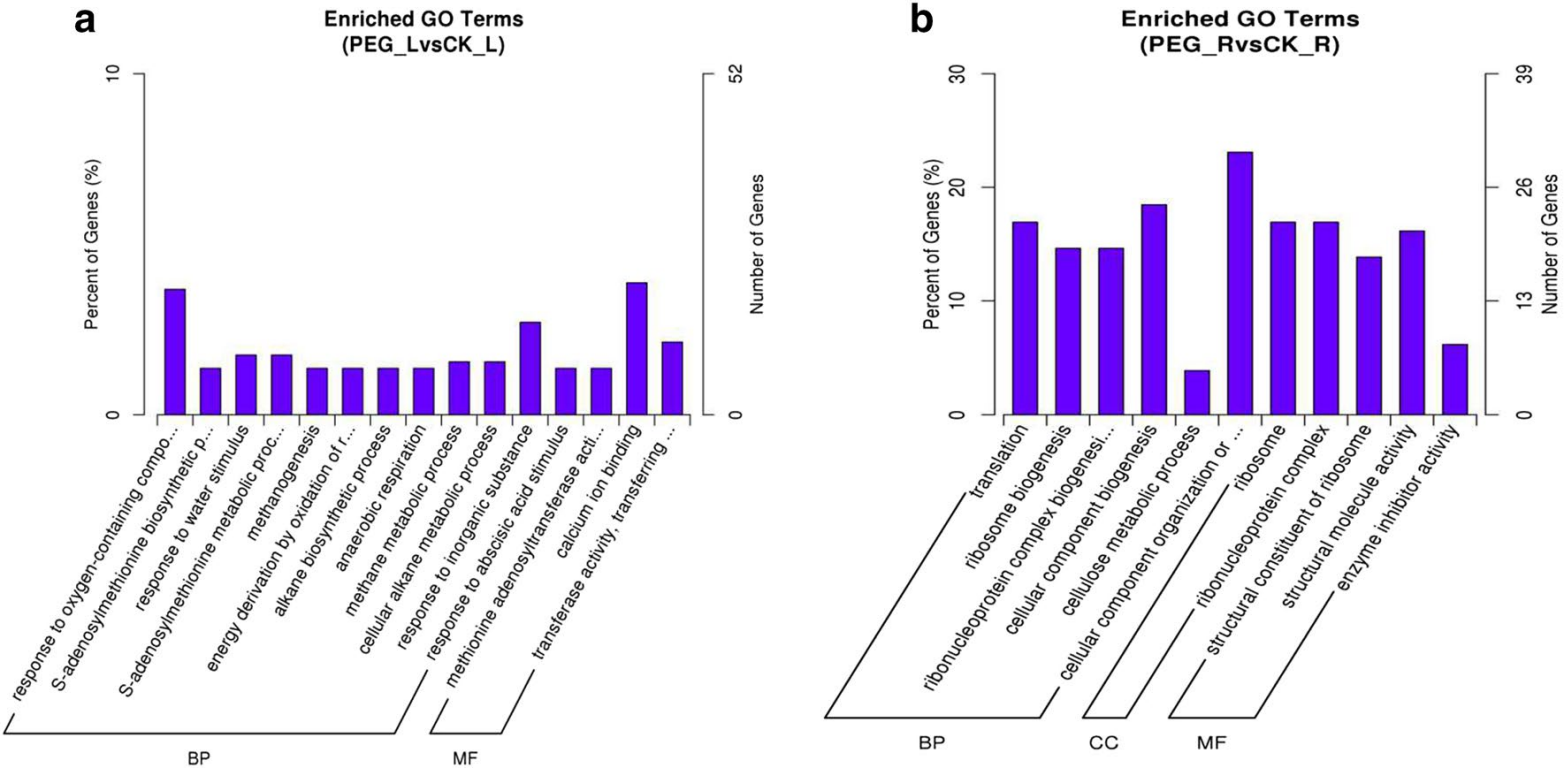
GO enrich analysis of significantly differently expressed genes in leaves and roots under drought stress. BP, CC and MF indicate biological process, cellular component and molecular function. **a** Enrich GO terms of significantly differently expressed genes in leaves under PEG treatment (PEG_L) and control group (CK_L). **b** Enrich GO terms of significantly differently expressed genes in roots under PEG treatment (PEG_R) and control group (CK_R)

Differentially expressed genes from leaves and roots were annotated using the GO database. Clearly annotated genes belonged to three main categories (cellular component, molecular function and biological process) and the distributions of gene numbers for each category were shown in Fig. 3. Notably, the “cellular component” category was not found within the top 15 categories in leaves.

In this study, we focused on a group of genes known to be involved in stress responses (Table 2). Of 17 genes involved in responses to abiotic stimuli, nine genes are known to be involved to responses to water stimuli and 16 genes were differentially expressed in leaves; only one gene, designated Comp11967_c0, was differentially expressed in both leaves and roots. We also found that each gene responded to more than one abiotic stress. This result indicated that relatively conserved mechanisms may be shared by sunflower responses to various abiotic stresses.

**Table 2:** Different expressed genes response to stress

Gene id	Annotation	Leaf			Root			Response to stress
		PEG-readcount	CK	pval	PEG	CK	pval	
Comp11967_c0	Dehydrin-like protein	10,175.3	1617.8	4.44E−08	120.8	1.9	7.27E−05	Water stimulus, chemical stimulus
Comp12003_c1	*H. annuus* mRNA for dehydrin related protein	5070.93	887.9	1.47E−07				Water stimulus, chemical stimulus
Comp14403_c0	NAC domain-containing protein	358.3	111.8	1.70E−06				Water deprivation, chemical stimulus, hormone stimulus, endogenous stimulus, organic substance
Comp17955_c0	Indole-3-acetic acid amido synthetase activity	33.43	5.4	1.64E−05				Hormone stimulus, endogenous stimulus, organic substance, chemical stimulus, radiation
Comp21257_c0	*S*-Adenosylmethionine synthetase	712.53	116.3	2.27E−14				Cold, temperature stimulus
Comp22725_c0	Galactinol synthase 2	31.43	6.9	0.0004				Abscisic acid stimulus, cold, oxidative stress, water deprivation, salt stress
Comp25287_c0	Aldehyde dehydrogenase family 7 member A1	2745.1	525.1	5.97E−13				Endogenous stimulus, salt, chemical stimulus, water stimulus, osmotic stress, organic substance, desiccation
Comp26183_c0	CBL-interacting serine/threonine-protein kinase	587.5	226.9	3.16E−05				Osmotic stress, salt, chemical stimulus, water stimulus,
Comp30219_c0	Glycosyl transferase family	1270.1	363.5	8.76E−08				Endogenous stimulus, salt, oxidative stress, chemical stimulus, temperature stimulus, water stimulus, organic substance, cold, osmotic stress
Comp305249_c0	DNA binding	0.3	10.6	0.0258				Radiation
Comp31776_c0	ATP-dependent Clp protease ATP-binding subunit ClpB	755.1	289.3	3.34E−05				Hydrogen peroxide, oxidative stress, chemical stimulus, temperature, light stimulus, reactive oxygen species, radiation
Comp32945_c0	Galactinol synthase 3	1420.8	461.4	0.0004				Chemical stimulus, water deprivation, temperature stimulus, cold
Comp34774_c0	Dehydrin	82412.6	5573.7	9.12E−29				Chemical stimulus, water stimulus
Comp35427_c0	Calcium-binding protein CML	1744.4	383.7	0.0003				Organic substance, endogenous stimulus, hormone stimulus, chemical stimulus, temperature stimulus, radiation
Comp36183_c0	Root phototropism protein 2	1014.1	2130.7	0.0455				Radiation
Comp39610_c0	Protein serine/threonine kinase activity	262.8	59.8	1.45E−08				Osmotic stress, endogenous stimulus, hormone stimulus, chemical stimulus, temperature stimulus, radiation
Comp42687_c0	DNAJ heat shock protein-like protein	216.9	66.0	2.04E−05				Hydrogen peroxide, oxidative stress, chemical stimulus, temperature stimulus, high light intensity, reactive oxygen species, heat, radiation

Gene id represent the sub-component serial number obtained by Trinity stitching

To identify the biological pathways represented within the DGE libraries, all annotated genes were mapped using the KEGG database to identify significantly enriched genes involved in metabolic or signal transduction pathways. Subsequently, 409 and 119 DEGs were assigned to 105 and 47 KEGG pathways in leaves and roots for drought, respectively. The top 20 pathways among all of the KEGG annotations of leaves and roots are listed in Table 3. The top KEGG pathways which contained the highest number of DEGs in leaves and roots were metabolic pathways, biosynthesis of secondary metabolites, plant hormone signal transduction, ribosome and RNA transport.

**Table 3:** Top 20 KEGG pathways of different expression genes in leaves and roots

Organs	Term	Gene number	Background number	P-value	Corrected P-value	Hyperlink
Leaves	Cysteine and methionine metabolism	15	164	7.70E−07	0.000141601	http://www.genome.jp/kegg-bin/show_pathway?ko00270
	Plant hormone signal transduction	19	320	1.77E−05	0.001632768	http://www.genome.jp/kegg-bin/show_pathway?ko04075
	Photosynthesis—antenna proteins	6	37	7.20E−05	0.00441311	http://www.genome.jp/kegg-bin/show_pathway?ko00196
	Other glycan degradation	3	23	0.009632561	0.338207262	http://www.genome.jp/kegg-bin/show_pathway?ko00511
	Porphyrin and chlorophyll metabolism	5	69	0.010937551	0.338207262	http://www.genome.jp/kegg-bin/show_pathway?ko00860
	Biosynthesis of secondary metabolites	43	1546	0.011028498	0.338207262	http://www.genome.jp/kegg-bin/show_pathway?ko01110
	Cyanoamino acid metabolism	4	56	0.02325574	0.523505194	http://www.genome.jp/kegg-bin/show_pathway?ko00460
	Valine, leucine and isoleucine degradation	5	85	0.024892835	0.523505194	http://www.genome.jp/kegg-bin/show_pathway?ko00280
	Biosynthesis of amino acids	16	479	0.025606232	0.523505194	http://www.genome.jp/kegg-bin/show_pathway?ko01230
	Metabolic pathways	70	2916	0.033671031	0.553343443	http://www.genome.jp/kegg-bin/show_pathway?ko01100
	Ascorbate and aldarate metabolism	4	66	0.039317106	0.553343443	http://www.genome.jp/kegg-bin/show_pathway?ko00053
	Pantothenate and CoA biosynthesis	3	41	0.04494819	0.553343443	http://www.genome.jp/kegg-bin/show_pathway?ko00770
	Carotenoid biosynthesis	3	41	0.04494819	0.553343443	http://www.genome.jp/kegg-bin/show_pathway?ko00906
	Lysine degradation	3	41	0.04494819	0.553343443	http://www.genome.jp/kegg-bin/show_pathway?ko00310
	Beta-Alanine metabolism	4	69	0.04510952	0.553343443	http://www.genome.jp/kegg-bin/show_pathway?ko00410
	Novobiocin biosynthesis	1	6	0.110953484	1	http://www.genome.jp/kegg-bin/show_pathway?ko00401
	Chlorocyclohexane and chlorobenzene degradation	1	6	0.110953484	1	http://www.genome.jp/kegg-bin/show_pathway?ko00361
	Phosphonate and phosphinate metabolism	1	7	0.128212836	1	http://www.genome.jp/kegg-bin/show_pathway?ko00440
	Styrene degradation	1	7	0.128212836	1	http://www.genome.jp/kegg-bin/show_pathway?ko00643
	Nitrogen metabolism	3	65	0.13220423	1	http://www.genome.jp/kegg-bin/show_pathway?ko00910
	Terpenoid backbone biosynthesis	3	67	0.141100694	1	http://www.genome.jp/kegg-bin/show_pathway?ko00900
	Benzoate degradation	1	8	0.14513793	1	http://www.genome.jp/kegg-bin/show_pathway?ko00362
Roots	Ribosome	16	979	0.000125245	0.023045141	http://www.genome.jp/kegg-bin/show_pathway?ko03010
	RNA transport	6	257	0.003419288	0.267326383	http://www.genome.jp/kegg-bin/show_pathway?ko03013
	Biotin metabolism	2	22	0.006784043	0.267326383	http://www.genome.jp/kegg-bin/show_pathway?ko00780
	Hippo signaling pathway—fly	3	75	0.008823808	0.267326383	http://www.genome.jp/kegg-bin/show_pathway?ko04391
	Arginine and proline metabolism	4	147	0.00971839	0.267326383	http://www.genome.jp/kegg-bin/show_pathway?ko00330
	Adherens junction	3	79	0.010167104	0.267326383	http://www.genome.jp/kegg-bin/show_pathway?ko04520
	Regulation of actin cytoskeleton	4	153	0.011134171	0.267326383	http://www.genome.jp/kegg-bin/show_pathway?ko04810
	Biosynthesis of unsaturated fatty acids	3	83	0.011622886	0.267326383	http://www.genome.jp/kegg-bin/show_pathway?ko01040
	Tight junction	3	94	0.016,217,849	0.331,564,911	http://www.genome.jp/kegg-bin/show_pathway?ko04530
	Hippo signaling pathway	3	110	0.024479743	0.438901021	http://www.genome.jp/kegg-bin/show_pathway?ko04390
	Focal adhesion	3	113	0.026238648	0.438901021	http://www.genome.jp/kegg-bin/show_pathway?ko04510
	Fatty acid biosynthesis	2	49	0.031,291,426	0.450,398,707	http://www.genome.jp/kegg-bin/show_pathway?ko00061
	Phagosome	4	211	0.031821648	0.450398707	http://www.genome.jp/kegg-bin/show_pathway?ko04145
	Two-component system	2	52	0.03,490,051	0.458,692,412	http://www.genome.jp/kegg-bin/show_pathway?ko02020
	Benzoate degradation	1	8	0.044298565	0.543395733	http://www.genome.jp/kegg-bin/show_pathway?ko00362
	Nitrogen metabolism	2	65	0.052231368	0.600660726	http://www.genome.jp/kegg-bin/show_pathway?ko00910
	Fatty acid metabolism	3	153	0.055894477	0.60497552	http://www.genome.jp/kegg-bin/show_pathway?ko01212
	Arachidonic acid metabolism	1	25	0.132,075,196	1	http://www.genome.jp/kegg-bin/show_pathway?ko00590
	Alanine, aspartate and glutamate metabolism	2	114	0.135824158	1	http://www.genome.jp/kegg-bin/show_pathway?ko00250
	Pentose and glucuronate interconversions	2	124	0.155261816	1	http://www.genome.jp/kegg-bin/show_pathway?ko00040

### Validation of DGE data by qRT-PCR

**Fig. 4:**
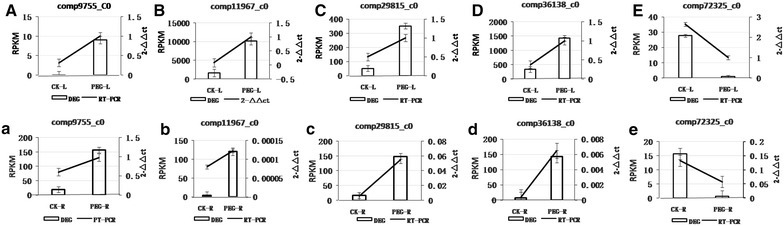
Comparison of genes expression profiles determined by qRT-PCR and DEG sequencing. Bars represent the standard deviations of three replicates. **A, a** Comparison of the expression of comp9755_c0 in leaves and roots determined by qRT-PCR and DEG sequencing, respectively. **B, b** Comparison of the expression profile of comp11967_c0 in leaves and roots determined by qRT-PCR and DEG sequencing, respectively. **C, c** Comparison of the expression profile of comp29815_c0 in leaves and roots determined by qRT-PCR and DEG sequencing, respectively. **D, d** Comparison of the expression profile of comp36138_c0 in leaves and roots determined by qRT-PCR and DEG sequencing, respectively. **E**, **e** Comparison of the expression profile of comp72325_c0 in leaves and roots determined by qRT-PCR and DEG sequencing., respectively

To evaluate the validity of DGE sequencing and to further analyze the patterns of differential gene expression, five genes were selected for detection using real-time PCR with gene-specific primers (Additional file 1: Table S1) and the data were compared to DEG sequence data. The results demonstrate that both in leaves and roots, four genes, comp9755_c0, comp11967_c0, comp29815_c0, comp36138_c0, were up-regulated after PEG treatment, but the expression of comp72325_c0 was significantly decreased during drought stress. The results of qRT-PCR are consistent with the trend observed for DEG sequencing (Fig. 4). Comparison of data obtained from DEG sequencing analysis methods to data from qRT-PCR confirmed the reliability of the DEG sequencing method.

## Discussion

Numerous studies have shown that adaptation mechanisms of plants in response to drought make up a complex metabolic regulatory network based on the expression of many genes. Although response mechanisms to various stresses are mainly conserved, specific plants often possess some of their own unique response mechanisms. Therefore, expression profiling studies of sunflower at the transcriptional level may benefit from knowledge of molecular drought tolerance mechanisms for other plants.

Before the large-scale application of high-throughput sequencing, cDNA microarrays were the traditional method for conducting gene expression profiling. Using cDNA microarrays, a number of drought response genes have been identified in *Arabidopsis thaliana* (Shinozaki et al. 2007), rice (Wang et al. 2007), maize (Zheng et al. 2006) and other crops. However, accurate expression profiling could only be conducted using a clear genome sequence to guide comparison of samples with identical genetic backgrounds for various conditions. Since no complete genome sequence exists for sunflower, cDNA microarray analysis could only be performed using the genome sequence of another crop. However, differing genetic backgrounds between sunflower and reference crops often caused high analytical uncertainty, generating erroneous results. In contrast, the sequences of unigenes obtained using high-throughput sequencing reflected objective changes in actual gene expression, but later annotation of DEGs was limited by the number of available reference sequences. The results of this study showed that DGE sequencing can achieve near complete coverage of the entire genome sequence with reliable and reproducible sequencing results under various conditions. However, we also found that the lack of genome sequence information may limit gene annotation, subsequently influencing in-depth mining and selection of candidate genes.

Of the total 1074 DEGs identified in this study, nine unigenes were associated with water stress, as demonstrated using annotation through sequence alignment. These DEGs included three dehydrin-like genes, one NAC transcription factor, two galactosidase family genes, one aldehyde dehydrogenase family gene, one CBL-interacting serine/threonine-protein kinase gene and one glycosyltransferase gene. Many researchers have observed a positive correlation between the expression and accumulation of plant dehydrin genes and plant resistance to abiotic stress (Hu et al. 2010). Meanwhile, the NAC transcription factor (Fang et al. 2015), galactosidase family gene (Santos et al. 2015), aldehyde dehydrogenase family gene (Singh et al. 2013), calcineurin-B-like (CBL)-interacting serine/threonine protein kinase gene (Chen et al. 2012) and glycosyltransferase gene (Tognetti et al. 2010) have all been shown to play important roles in response to abiotic and biotic stresses. Although 321 DEGs identified in this work could not be annotated, this study provides a rich set of candidate genes for further study of sunflower responses to drought stress. Although the functions and molecular mechanisms of these stress genes have not yet been fully elucidated, powerful genetic engineering methodologies, such as RNA interference, gene overexpression and transgenic technologies hold promise for defining their roles in sunflower drought tolerance.

## Conclusions

A total of 62,252 unigenes were obtained by transcript profiling sequencing in this study. Under drought stress, 876 and 269 DEGs were identified by DGE sequencing in sunflower leaves and roots, respectively. A greater number of up-regulated genes than down-regulated genes were identified in both leaves and roots. By analyzing and annotating DEGs, we found 17 genes that play roles in sunflower response to abiotic stimuli and thus may have relevance to drought tolerance. Of these, nine genes may be associated with responses to water-related stimuli. To our knowledge, this study is the first high throughput sequencing study for gene expression profiling analysis of sunflower under drought stress. The results of this study demonstrate that the drought stress response in sunflower leaves and roots comprises a complex mechanism involving multiple metabolic pathways. These findings have enriched our understanding of sunflower drought tolerance and provide many candidate genes associated with drought resistance for future development of sunflower varieties better suited to growth in marginal lands.

## Additional files

**Additional file 1: Table S1.** The sequences of primers of qRT-PCR.

**Additional file 2: Table S2.** The annotation of unigenes obtianed by transcriptome sequencing.

**Additional file 3: Table S3.** Go enrichment of unigenes obtained by transcriptome sequencing.

**Additional file 4: Table S4.** The sequences of SSR primers.

## Abbreviations

BADH: betaine aldehyde dehydrogenase
cDNA: complementary deoxyribonucleic acid
CBL: calcineurin-B-like
CK: control check
DEGs: differentially expressed genes
DGE: digital gene expression
DNA: deoxyribonucleic acid
DREB: dehydration responsive element binding protein
FPKM: Fragments Per Kilobase of exon model per Million mapped reads
KEGG: Kyoto Encyclopedia of Genes and Genomes
mRNA: messenger ribonucleic acid
NAC: no apical meristem
NCBI: National Center for Biotechnology Information
PCR: polymerase chain reaction
PEG: polyethylene glycol
qPCR: real-time quantitative PCR detecting system
RNA: ribonucleic acid
RSEM: RNA-Seq by expectation maximization
SYBR: synergy brands
